# Anticancer Activity of Extremely Effective Recombinant L-Asparaginase from *Burkholderia pseudomallei*

**DOI:** 10.4014/jmb.2112.12050

**Published:** 2022-03-27

**Authors:** Doaa B. Darwesh, Yahya S. Al-Awthan, Imadeldin Elfaki, Salem A. Habib, Tarig M. Alnour, Ahmed B. Darwish, Magdy M. Youssef

**Affiliations:** 1 Department of Biology, Faculty of Science, Tabuk University, Tabuk 71491, Saudi Arabia; 2 Botany Department, Faculty of Science, Mansoura University, Mansoura 35516, Egypt; 3 Biochemistry Department, Faculty of Science, Tabuk University, Tabuk 71491, Saudi Arabia; 4 Medical Laboratory Technology Department, Faculty of Applied Medical Sciences, Tabuk University, Tabuk 71491, Saudi Arabia; 5 Zoology Department, Faculty of Science, Suez University, El Salam-1, Suez 43533, Egypt; 6 Biochemistry Division, Chemistry Department, Faculty of Science, Mansoura University, Mansoura 35516, Egypt; 7 Department of Biology, Faculty of Science, Ibb University, 70270 Ibb, Yemen

**Keywords:** L-asparaginase, leukemia, cloning, DNA, purification, characterization

## Abstract

L-asparaginase (E.C. 3.5.1.1) purified from bacterial cells is widely used in the food industry, as well as in the treatment of childhood acute lymphoblastic leukemia. In the present study, the *Burkholderia pseudomallei* L-asparaginase gene was cloned into the pGEX-2T DNA plasmid, expressed in *E. coli* BL21 (DE3) pLysS, and purified to homogeneity using Glutathione Sepharose chromatography with 7.26 purification fold and 16.01% recovery. The purified enzyme exhibited a molecular weight of ~33.6 kDa with SDS-PAGE and showed maximal activity at 50°C and pH 8.0. It retained 95.1, 89.6%, and 70.2% initial activity after 60 min at 30°C, 40°C, and 50°C, respectively. The enzyme reserved its activity at 30°C and 37°C up to 24 h. The enzyme had optimum pH of 8 and reserved 50% activity up to 24 h. The recombinant enzyme showed the highest substrate specificity towards L-asparaginase substrate, while no detectable specificity was observed for L-glutamine, urea, and acrylamide at 10 mM concentration. THP-1, a human leukemia cell line, displayed significant morphological alterations after being treated with recombinant L-asparaginase and the IC_50_ of the purified enzyme was recorded as 0.8 IU. Furthermore, the purified recombinant L-asparaginase improved cytotoxicity in liver cancer HepG2 and breast cancer MCF-7 cell lines, with IC_50_ values of 1.53 and 18 IU, respectively.

## Introduction

Bacterial L-asparaginase plays a vital role as a therapeutic enzyme in the treatment of acute lymphoblastic leukemia [1]. The L-asparaginase enzyme catalyzes the conversion of the amino acid L-asparagine to L-aspartic in addition to ammonia [2]. This reaction leads to exhaustion of L-asparagine from the blood of leukemia patients which leads to the death of cancer cells faster than normal cells [3]. The guideline behind the cytotoxic impact of L-asparaginase stems from the reality that the leukemic lymphoblastic tumor cells and other blood tumor cells are auxotrophic to L-asparagine and show little L-asparagine synthetase action for de novo production of L-asparagine [4]. In this manner, these tumor cells require the exogenous supply of L-asparagine for multiplication and survival [5, 6].

L-asparaginase has been categorized into three classes based on the homology of the basic structure. The first class is the bacterial type II, a periplasmic L-asparaginase that can hydrolyze both L-asparagine and L-glutamine, and the enzymes have been dubbed glutaminase–asparaginases (E.C. 3.5.1.38) [7]. The plant-type L-asparaginase, which bears no resemblance to the bacteria-type enzyme, is the second class of L-asparaginase [8]. *Rhizobium etli* belongs to the third class of L-asparaginase, which has no known homologs with other L-asparaginases [9]. Commercially existing L-asparaginase is available in three formulae, namely, L-asparagine native from *Escherichia coli* [2], a PEGylated form of L-asparaginase, and *Erwinia chrysanthemi* L-asparaginase [10, 11].

The first L-asparaginase has a 978 bp open reading frame that encodes a 326-amino-acid protein with a 37 kDa molecular weight. This L-asparaginase was shown to be thermostable, naturally dimeric, and glutaminase-free, with a km of 12 mM and optimum activity at pH 9.0 [12]. The second uncharacterized L-asparaginase consists of a 933 bp open reading frame encoding a unique L-asparaginase with no glutaminase activity that shares homology with archaeon L-asparaginase [13].

The cloning, expression, purification, and biochemical characterization of a novel glutaminase-free L-asparaginase from *Burkholderia pseudomallei* are described in this paper. Furthermore, the purified recombinant enzyme was tested on acute monocytic leukemia THP-1, liver cancer HepG2, and breast cancer MCF-7 cell lines, for cytotoxicity. The findings of this work support the need to find new sources of microbial L-asparaginase that do not have glutaminase activity and are effective in killing leukemia and cancer cells.

## Materials and Methods

### Chemicals

Chemicals of molecular biology and analytical reagent grade were utilized in this study. As needed, the water used was deionized.

### Bacterial Strains and Plasmid DNA

*Burkholderia pseudomallei* bacterial strain [14], *E. coli* DH5 strain, BL21 (DE3) strain, and pGEX-2T DNA plasmid were generously contributed by Dr. Picksley, S. M. Bradford University, UK.

### Conditions of Media and Growth

LB medium was prepared by dissolving 10 g bacto-tryptone, 5 g yeast extract, and 10 g NaCl in one liter of deionized water and autoclaving it. Twenty grams of agar was added to one liter of LB medium to make LB agar plates. A 100 g/ml ampicillin supplement was added to the LB media (LBA).

### Chromosomal and Plasmid DNA

Both chromosomal and plasmid DNA were extracted and purified as described by Sambrook *et al*. [15].

### Polyacrylamide and Agarose Gels Electrophoresis

The method of Laemmli [16] was utilized to perform sodium dodecyl sulfate-polyacrylamide gel electrophoresis (SDS-PAGE). Horizontal agarose gel electrophoresis was utilized to examine DNA according to the previous report [17].

### Restriction Enzyme Digestion

Restriction enzyme digestion of DNA was performed according to the manufacturer's instructions. Heating the process at 70°C for 15 min and adding 1/6 volume of DNA loading dye brought the digestion to a finish.

### Polymerase Chain Reaction (PCR)

To make the cloning of the *B. pseudomallei* L-asparaginase gene process easier, oligonucleotide DNA primers forward (5'CG**GGATCC**GTTACAATCGCGGCC3') and reverse (5'GCGCGGC**GGATCC**TACG GCCGCG3') were synthesized with a defined BamHI restriction site (underlined). The L-asparaginase gene from *B. pseudomallei* chromosomal DNA was amplified using DNA of the forward and the reverse primers developed in a frame. The PCR reaction was carried out in a total volume of 50 µl, containing 2.5 µl of each primer (50 ng/l), 2.5 µl (2 mM) deoxynucleoside triphosphate mix, 3 µl Mg^++^ ion (25 mM), 5 µl buffer (10 × buffer provided with the *pfu* DNA polymerase enzyme), 1 µl template DNA (0.1 ng), 5 µl dimethyl sulphoxide (DMSO) and the volume completed to 50 µl with autoclaved deionized water. Two drops of mineral oil were added to each reaction tube. For 4 min, the reaction mixture was incubated at 94°C. The following PCR cycle was run 30 times: denaturation at 94°C for 1 min, primer annealing at 55°C for 1 min, and DNA synthesis at 72°C for 2 min. The mixture was then stored at 4°C after a 4-min incubation at 72°C.

### Cloning the *Burkholderia pseudomallei* L-Asparaginase Gene into pGEX-2T DNA Plasmid

As previously described [15], the amplified L-asparaginase gene from *B. pseudomallei* chromosomal DNA by PCR was treated with BamHI restriction enzyme and purified using low melting point agarose. The BamHI restriction enzyme was used to linearize a plasmid pGEX-2T DNA vector that had been purified. Using calf intestinal alkaline phosphatase, the plasmid's 5' phosphate ends were removed. The BamHI restriction enzyme-digested L-asparaginase gene was ligated into a plasmid that had already been treated with BamHI restriction enzyme and calf intestinal alkaline phosphatase. The ligation mixture was transformed into competent *E. coli* DH5 cells, which were then plated onto LBA plates and incubated overnight at 37°C. To identify recombinant plasmids, individual colonies were analyzed using plasmid micro prep and restriction enzyme digestion. The full L-asparaginase gene is in-frame with the GST protein on the recombinant plasmid. The GST-L-asparaginase protein was expressed in *E. coli* BL21 (DE3) cells transformed with the DNA recombinant plasmid.

### Overexpression of the *B. pseudomallei* L-Asparaginase Protein Over Time

*E. coli* having the DNA recombinant plasmid was streaked onto LBA plates and incubated at 37°C overnight. A single colony was inoculated into 10 ml of LB broth supplemented with 100 g/ml ampicillin and cultured overnight in a shaking incubator at 37°C and 200 rpm. To inoculate 100 ml LBA media, overnight cultures were employed. The cultures were cultured at 37°C and 200 rpm until they reached a mid-logarithmic growth phase with an OD650 nm of 0.4-0.6, at which point 1 mM of isopropyl-1-thio-B-galacto-pyranoside (IPTG) was added. One ml samples were taken at various periods, and the cells were pelleted by centrifugation at 6,000 ×*g* for 5 min. Cells were then resuspended in 100 ml of 1X SDS gel loading buffer: 20% (v/v) glycerol, 0.2% (w/v) bromophenol blue, 4% (w/v) SDS, 100 mM Tris-HCl, pH 6.8, and 200 mM dithiothreitol (DTT), followed by boiling for 4 min, sonication three times for 5 sec, and SDS-PAGE analysis.

### *Burkholderia pseudomallei* L-Asparaginase Protein Purification

The purification of *B. pseudomallei* L-asparaginase protein was performed as previously described [2].

### 3D Structural Modeling, Phylogenetic Tree Construction, and Sequence Analysis of *Burkholderia pseudomallei* L-Asparaginase

The nucleotide sequence of *B. pseudomallei* L-asparaginase was analyzed and compared to previously deposited sequences in the database using the Basic Local Alignment Search Tool (BLASTn and BLASTp) provided by NCBI (https://www.ncbi.nlm.nih.gov/protein/1104534862) and aligned using the ClustalO and DNA Star programs. Dereeper *et al*. [18] utilized Phylogeny.fr Software (http://www.Phlyogeny.fr) to create the phylogenetic tree for *B. pseudomallei* L-asparaginase. Milburn *et al*. [19] utilized software from http://www.ebi.ac.uk/thornton-sev/databases/sas/ to perform sequence annotation for *B. pseudomallei* L-asparaginase. Following a template search against the Swiss-Model template library with BLAST and HHBlits, three-dimensional (3D) structure prediction and model construction were carried out. BLAST against the primary amino acid sequence present in the SMTL was used to find the *B. pseudomallei* L-asparaginase target sequence. A total of 43 templates were revealed, and the template quality was predicted using target-template alignment features. For model construction, the highest-quality template was chosen. ProMod3 was then utilized to create models based on the target-template alignment. The template was utilized to copy coordinates that were conserved between the target and the template. Finally, the QMEAN scoring function [20] was utilized to evaluate the global and per-residue model quality.

### Enzyme and Protein Assay

The enzyme activity of *B. pseudomallei* L-asparaginase was assessed in terms of the hydrolysis rate of L-asparagine in the reaction by measuring the amount of ammonia produced. First, 10 mM of L-asparagine dissolved in 50 mM Tris–HCl at pH 8.6 was added to the enzyme samples. The enzyme-substrate combinations were incubated for 10 min at 37°C before being stopped by adding 100 µl of 1.5 M TCA. The amount of ammonia emitted was estimated using Nessler's reagent [21] and an ammonium sulfate solution as a standard, after which the samples were centrifuged and used for ammonia estimation. An international unit (UI) of L-asparaginase is defined as the amount of enzyme necessary to release one micromole of ammonia per minute at saturating substrate concentration under the assay conditions [22]. The Bradford dye method was used to quantify protein content, employing BSA as a reference at a concentration of 0.5 g/ml [23].

### Effect of pH and Temperature on Enzyme Activity

The *B. pseudomallei* L-asparaginase enzyme activities were evaluated at the pH range of 6.0 to 10.0, and 100 mM Tris–HCl (pH 6.0–10.0) was employed as a buffer. The reactions were carried out in a temperature-controlled water bath at their optimal pH values and throughout a temperature range of 20 to 80°C to investigate the effect of temperature on pure L-asparaginase enzyme activity.

### Effect of Metal Ions, EDTA, and Reducing Agents

On the activity of the purified *B. pseudomallei* L-asparaginase, the effects of metal ions, ethylenediaminetetraacetic acid (EDTA), and reducing agents dithiothreitol (DTT) and 2-mercaptoethanol (2- C_2_H_5_SH) were investigated. Following the determination of enzyme activity, the purified enzyme was incubated for 15 min on ice with 1 mM and 5 mM of each agent individually. The residual activities of the purified recombinant enzyme were evaluated after adding EDTA at concentrations of 1 and 5 mM to the purified enzyme, followed by the addition of 500 µl of 15% trichloroacetic acid.

### Substrate Specificity

The purified enzymés substrate specificity was determined using the substrates L-asparagine, L-glutamine, urea, and acrylamide. The relative activities of these substrates were determined when they were used in place of L-asparagine at a concentration of 10 mM.

### In Vivo Study

Adult female Swiss mice weighing 22 ± 0.32 grams from Animal House Biological Products & Vaccines (VASERA) in Cairo, Egypt were used in the study. Before starting the experiment, the animals were kept in a clean cage for 2 weeks for adjustment. They were fed a standard diet and were free to drink water before being divided into 4 groups (8 animals each). All appropriate precautions and procedures used in this experiment were approved by the Animal Ethics Board of Mansoura University in Egypt. The first, second, and third groups received a single dosage of purified *B. pseudomallei* L-asparaginase at concentrations of 100, 1,000, and 5,000 IU, respectively. Blood samples were taken in EDTA-treated tubes after 4, 8, and 24 h and residual *B. pseudomallei* L-asparaginase activity was measured [24]. According to the manufacturer's recommendations, serum albumin, enzymes (AST, ALT), and lipid profile (cholesterol and triglyceride) were used to assess liver-associated plasma proteins and lipid profiles.

### Cell Culture and Cytotoxicity Test Using Alamar Blue and MTT Assay

The THP-1 cell line was offered by ATTC for this study. VACSERA, a holding business for biological products and vaccines in Cairo, Egypt, provided the HepG2 and the MCF-7 cell lines. THP-1 cells were grown in RPMI 1640 medium, which included 10% heat-inactivated fetal bovine serum, 1% glutamine, 100 U/ml penicillin, and 100 mg/ml streptomycin. On a 96-well plate, cells were seeded at a density of 10,000 cells/well before being treated with different amounts of purified *B. pseudomallei* L-asparaginase and incubated for 48 h at 37°C in 5% CO_2_. Untreated cells were seeded in the same circumstances as the treated cells, in a 20 mM potassium phosphate buffer (pH 7.5). Following incubation, each well received 10 µl of alamarBlue reagent (10% alamarBlue, Invitrogen, USA), and incubation was maintained at 37°C for another 4 h. The absorbance of the plates was measured at 570 nm for the plates and 600 nm for the reference using a microplate reader. The percentage of cell viability was expressed relative to the control cells after blank normalization [25]. Morphological changes in THP-1 cells were explored and documented using phase-contrast optical microscopy at a magnification of 40. The HepG2 and MCF-7 cell lines were cultured in DMEM high glucose media (4.5 g/l) supplemented with 10% FCS and 1%penicillin/streptomycin at 37°C and 5% CO_2_. For the 3-(4, 5-Dimethyl-2-thiazolyl)-2, 5-diphenyl-2H-tetrazolium bromide reagent (MTT) test, cells were seeded at a density of 10,000 cells/well in a 96-well plate. The media were replaced after 24 h with a new mixture containing different concentrations of *B. pseudomallei* L-asparaginase, which was cultivated for 48 h. The cells were incubated for 3 h at 37°C in 5% CO_2_ after being given MTT (5 mg/ml in 1 PBS). The cells were centrifuged and incubated in 100 µl of DMSO after incubation. After agitating the plates for 5 min, the absorbance was measured at 490 nm. For both treated and untreated cells, the proportion of viable cells was measured as control and plotted against *B. pseudomallei* L-asparaginase concentrations to calculate the IC_50_ [26].

### Statistical Analysis

For statistical analysis, GraphPad Prism 5 software was employed (GraphPad Software, Inc., USA). A two-tailed Student's t-test was used to compare two groups. Tukey's post hoc test for unpaired nonparametric variables was used to assess differences between groups when more than two were compared using a one-way test (ANOVA). Outliers having a Q of 1% were found using ROUT. The mean SEM or SD is calculated using data from at least two distinct studies and two replicates.

## Results

### *Burkholderia pseudomallei* L-Asparaginase Gene Identification and Sequence Analysis

**Fig. 1:**
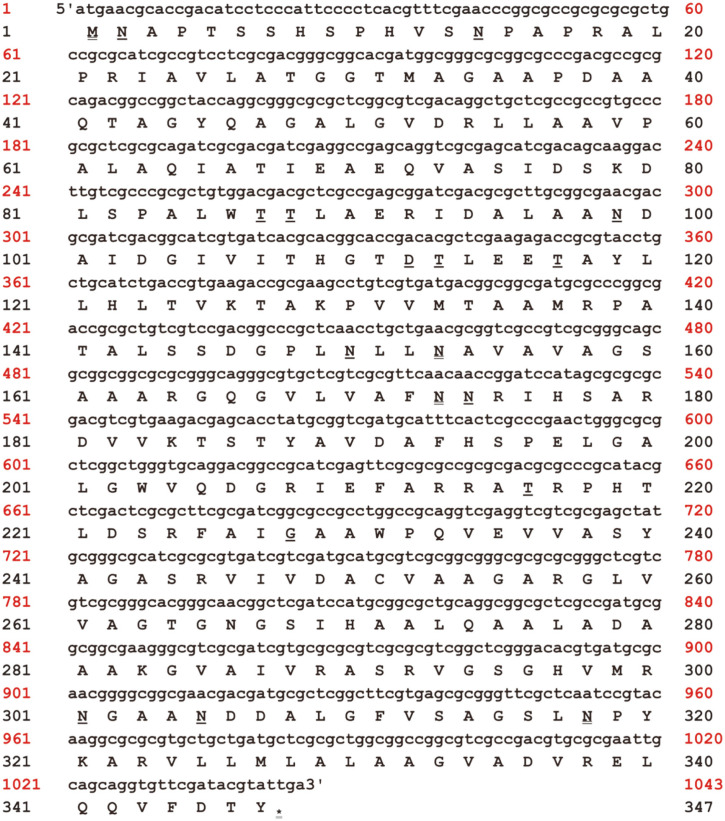
*Burkholderia pseudomallei* L-asparaginase nucleotide and deduced amino acid sequence. The Lasparaginase amino acid signature (residues Asparagine 153,173, 318, Threonine 113, 117, 216, 220, and Glycine 228) is displayed in bold underlining. The start codon (atg, Methionine) is highlighted with a bold double underline, and the asterisk denotes the stop codon (tga).

**Fig. 2:**
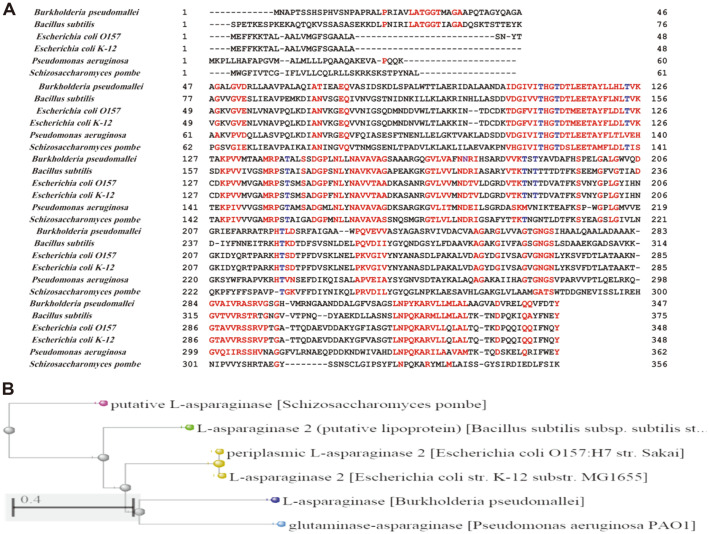
Pairwise alignment (**A**) and phylogenetic relationship (**B**) of *Burkholderia pseudomallei*, *Bacillus subtilis*, *Escherichia coli* *O157*, *Escherichia coli* *K-12*, *Pseudomonas aeruginosa*, and *Schizosaccharomyces pombe* L-asparaginase. Red asterisks show the conserved segment near the N-terminal end and the blue asterisks show the conserved threonine residues representing the catalytic triad threonine 113, 117, 124, 222 involved in catalysis (**A**). Maximum probability tree is based on GenBank-deposited full coding sequences (**B**).

A unique L-asparaginase (https://www.ncbi.nlm.nih.gov/protein/1104534862) was documented in the genome of *B. pseudomallei*, whose entire sequence was obtained and deposited in the GenBank database. The *B. pseudomallei* L-asparaginase gene has 1,041 base pairs and is coded for a protein with 347 amino acids, according to sequence analysis (Fig. 1). The Blast P program in the NCBI Blast server was utilized to compare the protein sequence of *B. pseudomallei* L-asparaginase to L-asparaginase from *Bacillus subtilis*, *Escherichia coli* *O157*, *Escherichia coli* *K-12*, *Pseudomonas aeruginosa* and *Schizosaccharomyces pombe*, and the results showed statistically significant high similarity scores (Table 1). *B.pseudomallei* 1041b (GenBank Accession No. ABA50799.1) had the highest percentage of sequence identity (99.71%) and *Neisseria meningitides* (28.1%) had the lowest percentage of sequence identity (GenBank Accession No. WP_002229812.1) (Table 1). Fig. 2A displays the alignment of the deduced amino acid sequence of *B. pseudomallei* L-asparaginase with *Bacillus subtilis*, *Escherichia coli* O157, *Escherichia coli* K-12, *Pseudomonas aeruginosa* and *Schizosaccharomyces pombe* representative members of the L-asparaginase family. The phylogenetic tree (Fig. 2B) was built using the neighborhood-joining approach based on the *L-asparaginase* amino acid sequence of *E. coli* O157, *Escherichia coli* K-12, *B. subtilis*, *P. aeruginosa* and *Schizosaccharomyces pombe* shared phylogenetic similarities with *B. pseudomallei* L-asparaginase, but migrated to other clusters away from other bacterial species, such as *E. coli* *O157*, *E. coli* *K-12* and *P. aeruginosa* (Fig. 2B), demonstrating L-asparaginase divergence.

### 3D Structure Prediction for *Burkholderia pseudomallei* L-Asparaginase

**Fig. 3:**
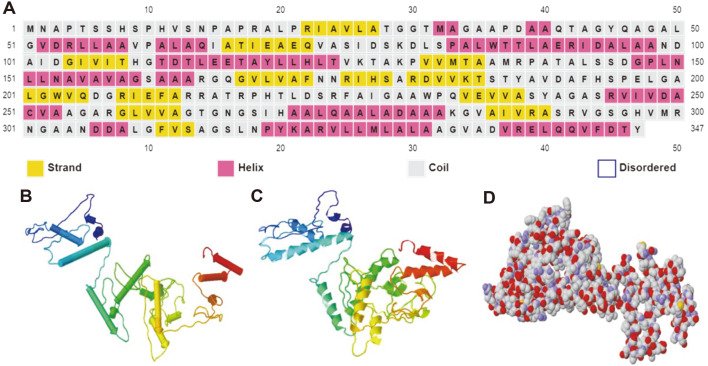
(**A**) Amino acid sequence alignment of *Burkholderia pseudomallei* L-asparaginase. Yellow boxes (- strands) and pink boxes (-helices) and gray boxes (-coil) represent secondary structural components. (**B**) A cartoon model of the expected 3D structure of *Burkholderia pseudomallei* L-asparaginase. The secondary structurés components are colored red for -helices, yellow for -strands, and green for twists and coils. (C-D) *Burkholderia pseudomallei* L-asparaginase predicted 3D structure -helices are blue, -strands are red, and coils are cyan in this cartoon representation of a homodimer.

*B. pseudomallei* L-asparaginases sequence explanation and secondary structural motif elements (Fig. 3A) revealed several intriguing conserved traits. To begin, a signature for L-asparaginase was revealed, which consisted of conserved invariant amino acid residues including Asparagine 153,173, 301, 305, 318, Threonine 113, 117, 222, and Glycine 228, which were involved in substrate (Asparagine) recognition, binding, and catalysis. The secondary structure of *B. pseudomallei* L-asparaginase (Fig. 3A) was expected to have a maximum of 8 helical structures (35%) and 11 strands (25.6%), as well as a large number of sites for favorable coil and turn formation. The predicted 3D structure of *B. pseudomallei* L-asparaginase was a homodimer with 8-helices and 11-strands (Figs. 3B-3D), which was fairly close to that of h L-asparaginase3. The structure was determined to have the conserved C-terminal amino acid residues G_284_VAIVRASRVG_294_ seen in *B. subtili*, *E. coli* *O157*, *E. coli* *K-12*, *P. aeruginosa*, and L-asparaginases (Fig. 2A). In the presence of high threonine concentrations, (Fig. 3A) _108_THGT_111_ has been found to play an important role in the cleavage reaction and autoactivation of *B. pseudomallei* L-asparaginase.

### Time Course and Expression of *Burkholderia pseudomallei* L-Asparaginase Polypeptide

**Fig. 4:**
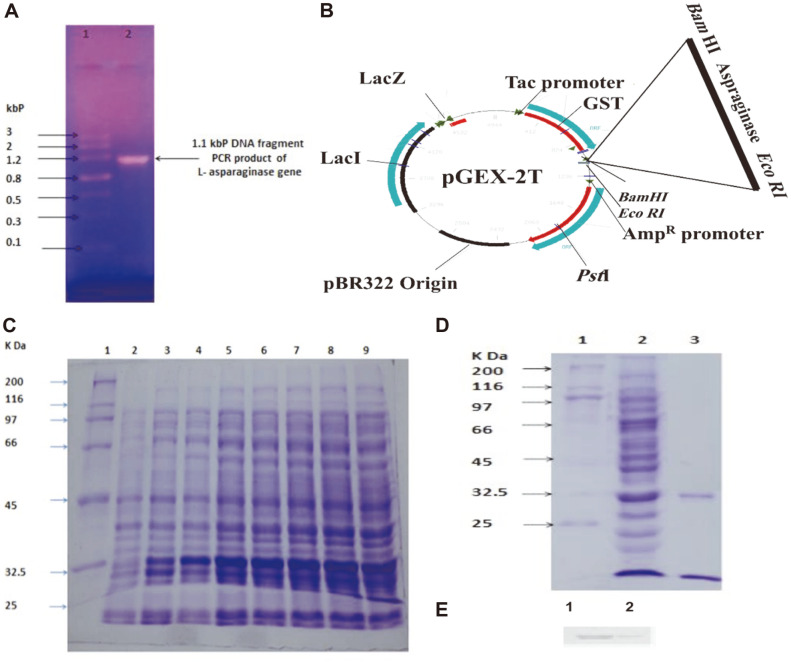
(**A**) The PCR product of the 1.1 kbp DNA fragment of the L-asparaginase gene of *Burkholderia pseudomallei*. The DNA fragment was analyzed on a 1.2% TAE agarose gel. Lane 1: DNA marker (Gel pilot wide range ladder 100 -Qiagen). Lane 2: 1.1 kbp DNA fragment PCR product of L-asparaginase gene. (**B**) Schematic diagram of the recombinant *Burkholderia pseudomallei* L-asparaginase overexpressions construct. The Lasparaginase gene was cloned downstream of the Tac promoter in the pGEX-2T DNA expression vector, which also contained the genes for lacI and lacZ repressors, pBR322 origin, and ampicillin resistance. (**C**) Induction time course for overexpression of L-asparaginase protein. Early to the mid-log culture of *E. coli* BL21 with Lasparaginase recombinant plasmid was induced at time 0 h with IPTG at a final concentration of 1 mM and samples were taken and analyzed by 10% SDS-PAGE gel at times indicated. Lane 2-8: protein marker, Lane 1: Sigma SD6H2 (MW 25,000-200,000 kDa). (**D**) The purification profile of the L-asparaginase protein on SDSPAGE. Lane 1: protein marker, Lane 2: *E. coli* L-asparaginase crude extract, Lane 3: Glutathione S sepharose 4B column-eluted L-asparaginase. (**E**) Western blot analysis with anti-GST antibody. Lane 1: crude extract, Lane 2: purified L-asparaginase.

With the specified forward and reverse oligonucleotides primers, the L-asparaginase gene was amplified by PCR from *B. pseudomallei* chromosomal DNA, providing the expected 1.1 kbp DNA product (Fig. 4A) including the 1,041 bp L-asparaginase gene with flanking DNA. In the pGEX-2T DNA plasmid, the PCR product was ligated into the BamHI restriction site under the control of the IPTG-inducible Tac promoter and the lacI repressor (Fig. 4B). The L-asparaginase gene was in-frame and oriented correctly concerning the plasmid *tac* promoter in the generated plasmid, L-asparaginase.

The appearance of the putative induction of *B. pseudomallei* L-asparaginase polypeptides through time is represented in Fig. 4C. At time 0 h, 1 mM IPTG was added to *E. coli* transformed with the recombinant plasmid, and samples were obtained every 1 h. After 2 h of IPTG induction, overproduction of the *B. pseudomallei* L-asparaginase was evident (Fig. 4C, lane 5), and peak expression was obtained after 5 h (Fig. 4C, lane 8). The greatest expression of the L-asparaginase polypeptide occurred after 5 h of IPTG induction.

The coding sequence of *B. pseudomallei* L-asparaginase was cloned and produced in *E. coli* BL21 (DE3) pLysS under the control of the T7 promoter of the pGEX-2T DNA plasmid. The Fast Flow glutathione S sepharose 4B column was utilized to purify the GST fusion recombinant L-asparaginase, The glutathione S sepharose 4B column matrix was utilized to bind the recombinant protein, which was then eluted from the column with buffer containing 10 mM reduced glutathione. For the pure recombinant *B. pseudomallei* L-asparaginase, SDS-PAGE examination revealed a single band of 33,660 Da (Fig. 4D). Western blot analysis with anti-GST monoclonal antibody confirmed the identity of the purified recombinant enzyme, and a single unique band of the correct size was observed, (Fig. 4E). The purified enzyme had a specific activity of 15,001.67 U/mg protein, and the purification fold of the purified recombinant enzyme was 7.26, resulting in a total yield of 16.01% (Table 2).

### Characterization of *Burkholderia pseudomallei* L-Asparaginase

**Fig. 5:**
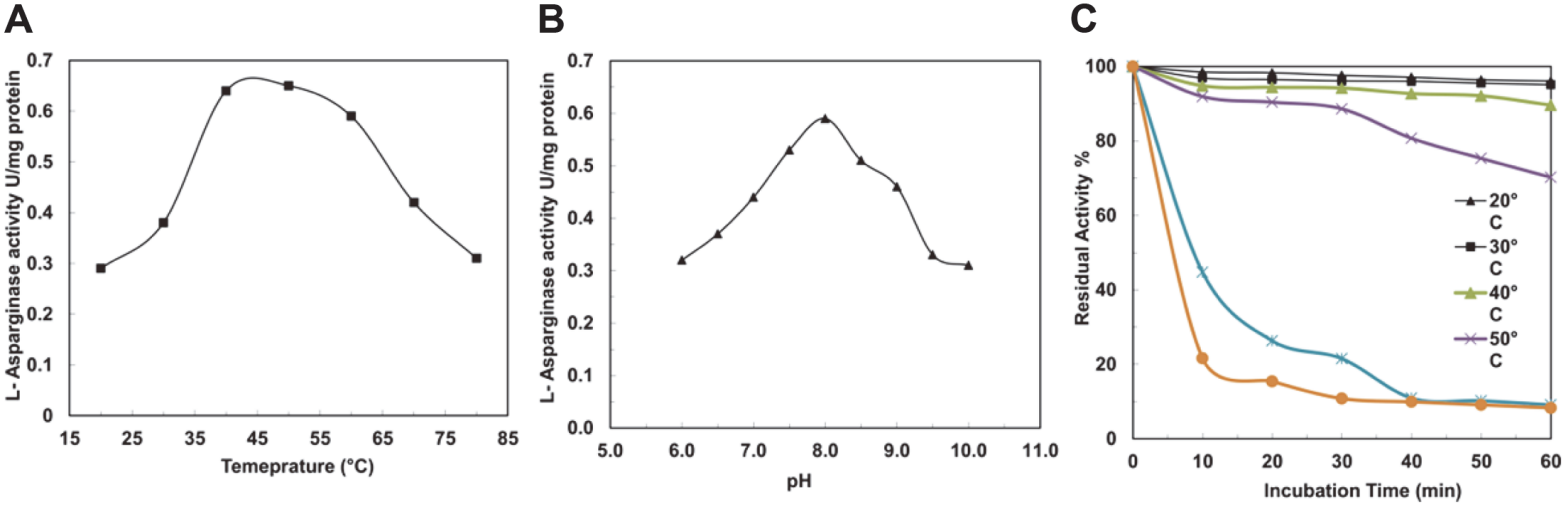
The purified *Burkholderia pseudomallei* L-asparaginase at its optimal temperature (**A**), pH (**B**), and thermostability (C). The results are expressed as the means ± SD from three independent experiments.

The pure *B. pseudomallei* L-asparaginase enzyme was active at temperatures ranging from 37 to 55°C, with an optimal temperature of 50°C (Fig. 5A). When it came to the appropriate pH, the purified enzyme performed best at pH 8.0 (Fig. 5B). The thermostability of the purified recombinant enzyme was also tested, and it was revealed that the enzyme has a wide range of thermostabilities between 30 and 60°C. The purified *B. pseudomallei* L-asparaginase was found to be thermostable for 60 min at 30°C with 95.1% residual activity, while residual activity was reduced after 60 min at 40°C and 50°C (89.6% and 70.2%, respectively) (Fig. 5C).

### Substrate Specificity of *Burkholderia pseudomallei* L-Asparaginase

The absence of glutaminase activity is a major advantage for using L-asparaginase in the treatment of ALL. Various reaction substrates were investigated to determine the substrate specificity of *B. pseudomallei* L-asparaginase. At a concentration of 10 mM, the purified recombinant enzyme displayed the maximum activity and specificity towards the reaction substrate L-asparagine, with no measurable activity towards the other substrates L-glutamine, urea, or acrylamide.

### Effect of Metal Ions, EDTA, and Reducing Agents

Sulfate and chloride metal ions, as well as reducing agents, were studied (Table 3). At a concentration of 1 mM, both KCl and NaCl increased L-asparaginase activity, whereas ZnCl_2_, CuCl_2_, HgCl_2_, MgCl_2_, and CaCl_2_ inhibited it in the following order: HgCl_2_ > CaCl_2_ > CuCl_2_ > ZnCl_2_ > MgCl_2_. On the other hand, most of the examined metal ions in sulfate forms inhibited *B. pseudomallei* L-asparaginase activity. At 1 mM and 5 mM concentrations, reducing agents like DTT and 2-mercaptoethanol reduced the enzyme activity marginally (Table 3). The effect of the metal-chelating compound EDTA was also studied, and it was revealed that EDTA decreased the activity of *B. pseudomallei* L-asparaginase by 60.7 and 41.2%, respectively, at concentrations of 1 mM and 5 mM.

### In Vivo Study

**Fig. 6:**
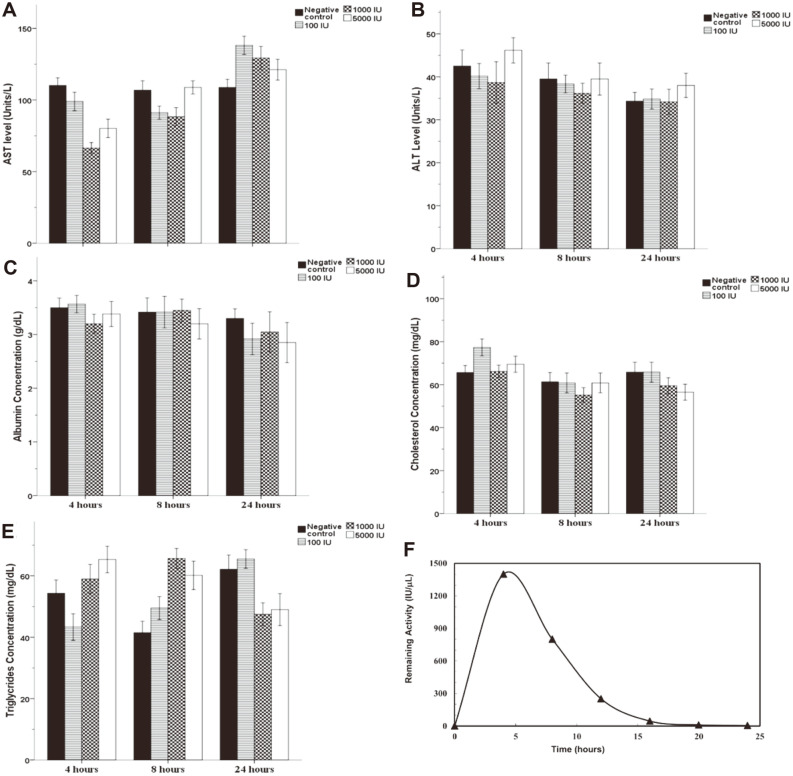
Effects of purified recombinant *Burkholderia pseudomallei* L-asparaginase on rat liver enzymes, AST (**A**), ALT (**B**), albumin (**C**), cholesterol (**D**), and triglyceride (**E**), at various time intervals ranging from 4 to 24 h after injection. (**F**) Purified *Burkholderia pseudomallei* L-asparaginase serum half-life in vivo. The results are expressed as the means ± SD from three independent experiments.

In vivo studies on rats given various concentrations of purified recombinant *B. pseudomallei* L-asparaginase as an acute dose (Figs. 6A-6E) revealed that even higher concentrations of L-asparaginase (5,000 IU) had no significant effects on hepatic enzymes AST (**A**), ALT (**B**), albumin (C), cholesterol, and triglycerides (D and E). The recombinant L-asparaginase activity was also investigated in rats given different concentrations of the enzyme ranging from 100 to 5,000 IU, and it was revealed that the L-asparaginase activity detected after 2 h in the animal group given 5,000 IU dramatically declined after 12 h to 5.6% of the original activity, while no enzymatic activities were detected in the groups given 100 and 1,000 IU (Fig. 6F). Renal clearance of the *B. pseudomallei* L-asparaginase, particularly at lower doses, could account for these findings.

### Cytotoxicity of Recombinant *Burkholderia pseudomallei* L-Asparaginase on Cell Lines

**Fig. 7:**
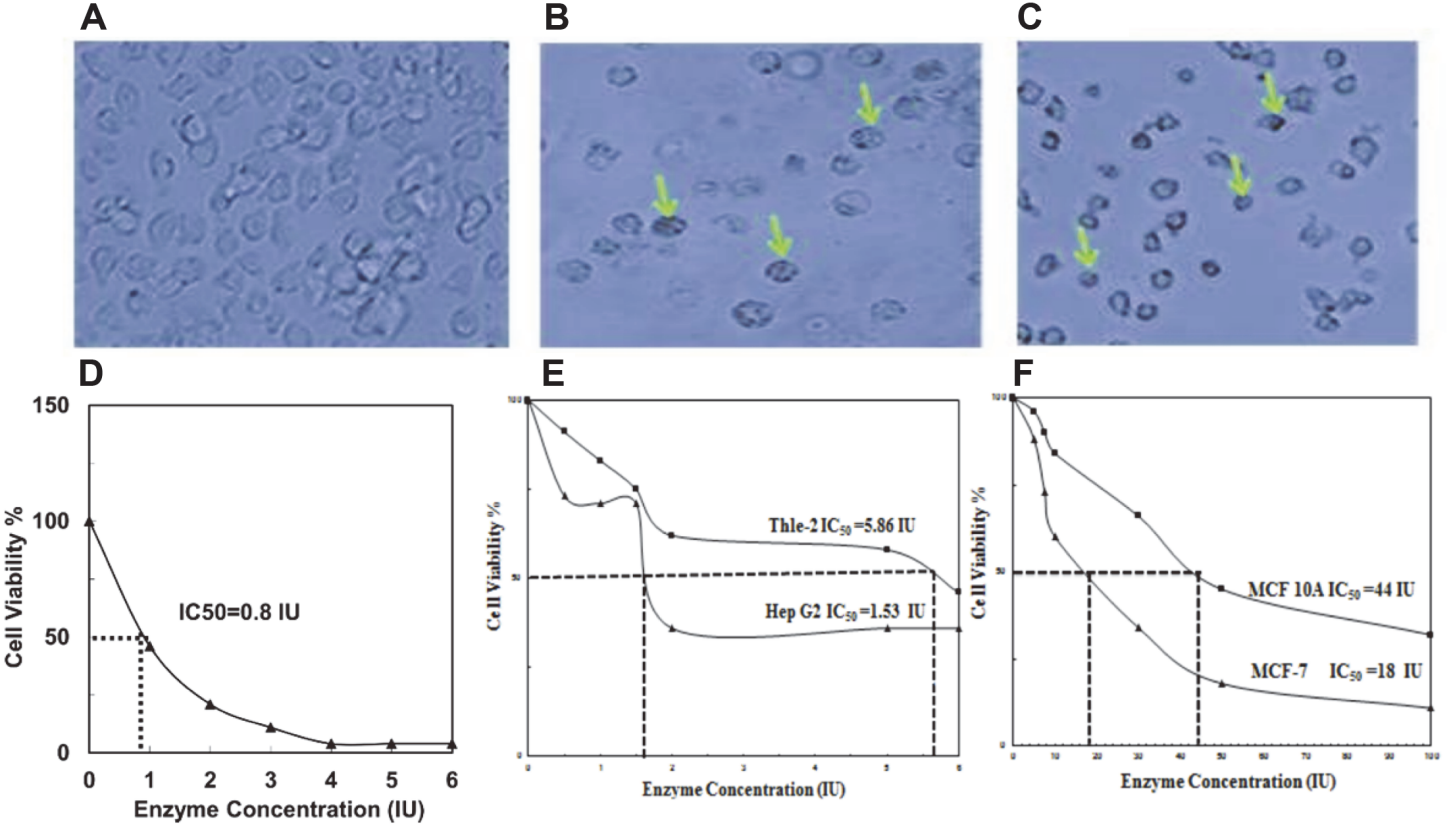
The shape of human leukemia THP-1 cells is altered by recombinant *Burkholderia pseudomallei* Lasparaginase. Purified recombinant L-asparaginase at a concentration of 1 IU was used to treat cells for 48 h THP-1 cells that had not been treated (**A**), paclitaxel-treated cells (**B**), and purified recombinant L-asparaginase-treated cells (**C**). The intracytoplasmic granules are indicated by green arrows. (**D, E**, and **F**) THP-1, HepG2, and MCF-7 cell lines are all killed by *Burkholderia pseudomallei* L-asparaginase. Different concentrations of *Burkholderia pseudomallei* L-asparaginase were utilized to treat cell lines for 48 h. The percentage of cell viability was calculated using alamarBlue and MTT tests. The IC_50_ of *Burkholderia pseudomallei* L-asparaginase for THP-1, HepG2, and MCF-7 was calculated. The results are expressed as the means ± SD from three independent experiments.

To investigate the effects of purified recombinant *Burkholderia pseudomallei* L-asparaginase on the human leukemia cell line THP-1, different concentrations of the pure *B. pseudomallei* L-asparaginase were utilized to treat the cells. Significant morphological alterations were found after 48 h of therapy, according to our findings (Figs. 7A and 7C). With the production of intra-cytoplasmic granules and apoptotic bodies, the enzyme-treated cells were reduced in number, size, and shrinkage. These morphological changes were also detected in cells treated with paclitaxel at a dose of 20 µM as a positive control (Fig. 7B) when compared to untreated cells (Fig. 7A). Cell viability and cell death appear to be inhibited by these extreme changes in cell shape. The alamarBlue assay was used to test the effect of the recombinant *B. pseudomallei* L-asparaginase on THP-1 cell viability, and the results showed that the recombinant L-asparaginase decreased cell viability in a dose-dependent manner, with an IC_50_ of 0.8 IU (Fig. 7D).

The MTT assay was used on normal liver cell line THLE-2 and liver cancer cell line HepG2 to assess the anticancer and cytotoxicity effects of recombinant *B. pseudomallei* L-asparaginase. The IC_50_ values of recombinant L-asparaginase against normal liver cell line THLE-2 and liver cancer cell line HepG2 were found to be 5.9 and 1.53 IU, respectively (Fig. 7E). Moreover, the MTT test was performed on normal breast MCF 10A and breast cancer MCF-7 cell lines to investigate the anticancer and cytotoxicity effects of recombinant *B. pseudomallei* L-asparaginase. The IC_50_ values of recombinant L-asparaginase against normal breast MCF 10A and breast cancer MCF-7 cell lines were 44 and 18 IU, respectively (Fig. 7F).

## Discussion

Overproduction of economically important pharmaceutical enzymes like L-asparaginase has been achieved using recombinant DNA technology in a different bacterial host. This enzyme is controlled by a number of genetic elements found in various bacterial genera. L-Asparaginase is found in an operon with L-asparaginase B, which encodes L-asparaginase, in Bacillus. The expression of the L-asparaginase AB operon is inhibited by L-asparaginase R, and the activity of L-asparaginase R is thought to be regulated by asparagine or aspartate. The gene for L-asparaginase was cloned, overexpressed, and characterized from a non-pathogenic strain of *B. pseudomallei*. The Blast P program in the NCBI Blast server was utilized to compare the protein sequence of *Burkholderia pseudomallei* L-asparaginase to L-asparaginase from *Bacillus subtilis* [27], *Escherichia coli* *O157* [28], *Escherichia coli* *K-12* [29], *P. aeruginosa* [30] and *Schizosaccharomyces pombe* [31], and the results showed statistically significant high similarity scores (Table 1). Sequence annotation by structure revealed that the *Burkholderia pseudomallei* L-asparaginase lacks the L-glutaminase active site signature, which is found in most microbial L-asparaginase, including *E. coli* and *E. chrysanthemi*. These L-asparaginases have dual activities against both the reaction substrates, L-asparagine and L-glutamine, and typically account for 2–10% of their L-asparaginase activity [32]. Because of the development of immunogenicity and cytotoxicity associated with the treatment of acute lymphoblastic leukemia patients [33], this property of *B. pseudomallei* L-asparaginase is noted with high significance.

The 60 kDa lysophospholipase enzyme hydrolyzes lysophospholipids as well as L-asparagine. This enzyme is also related to *E. coli* type I and II L-asparaginase and belongs to the bacterial type family [34]. *E. coli* type I and II L-asparaginase is identical to this enzyme. Human L-asparaginase is a lysosomal aspartylglucosaminidase and a plant type L-asparaginase that removes carbohydrate groups connected to asparagine [35, 36]. Third, human L-asparaginase is h asparaginase3, a plant type L-asparaginase with high structural resemblance to *E. coli* type III L-asparaginase [37, 38].

In the presence of free amino acid glycine, this conserved region, _265_GNG_267_, is implicated in h asparaginase3 auto-cleavage, self-activation, and catalytic activity [39]. Four threonine residues, threonine_111, 113, 117, 124, 222_, were discovered in the catalytic triad of *B. pseudomallei* L-asparaginase, which are important and responsible for the catalytic activity towards the L-asparagine substrate.

The crucial and critical threonine residue is Thr_220_, which is not required for autocleavage but is required for catalysis because the Thr_217_ hydroxyl group acts as an activator for the hydroxyl group of Thr_220_ [33]. The Thr_219_ (in humans) and Thr_220_ (in *B. pseudomallei* L-asparaginase) residues are the third and fourth threonine residues in the catalytic triad of both h L-asparaginase3 and *B. pseudomallei* L-asparaginase. This conserved threonine residue, along with the nearby glycine moiety (Gly_202_ or _207_ or _228_), influences the movement of the glycine-rich region, which is a _206_DG_207_ loop at the N-terminal region of the L-asparaginase that changes the conformation between the cleavage and un-cleavage states. As a result, the catalytic mechanism for h asparaginase and *B. pseudomallei* L-asparaginase towards the L-asparagine substrate could be very similar. The mechanism begins with a nucleophilic attack on the carboxyl group of L-asparaginase by the Thr_220_ side chain, which is followed by the release of the amino group. A -amino group near the Asp_222_ side chain and the His_219_ carbonyl atom is also involved in the action. The oxyanion hole has been postulated to stabilize negatively charged tetrahedral intermediates [40]. Thr_220_ and His_219_ residues have been reported to be part of it. Surprisingly, the activity of isolated recombinant *B. pseudomallei* L-asparaginase was discovered.

The thermostable L-asparaginase from *Pyrobaculum calidifontis* was found to have an optimum temperature of at least 100°C and a pH of 6.5 [41]. The optimal pH and temperature for pure thermostable L-asparaginase from *Bacillus amyloliquefaciens* were 8.5 and 65°C, respectively [42]. This finding is significant because of the cytotoxicity associated with glutaminase activity, which is generally associated with *E. coli* and *E. chrysanthemi* L-asparaginase activity [32]. Furthermore, these findings corroborate the results of sequence annotation by structure, which demonstrated the absence of the L-glutaminase signature in *B. pseudomallei* L-asparaginase.

Treatment of acute lymphoblastic leukaemia patients with L-asparaginase is linked to hypertriglyceridemia [43], liver function, and hepatic transaminase impairment, as well as bilirubin and alkaline phosphatase increases [44]. In addition, increased hepatic transaminase, alkaline phosphatase, and bilirubin levels have been recorded in 30–60% of patients receiving L-asparaginase as part of multiagent therapy [45].

L-Asparaginase has been shown to have antileukemic and anticancer properties [46], but the effect of recombinant *B. pseudomallei* L-asparaginase on human leukemia and cancer cells has yet to be fully explored.

The purified recombinant *B. pseudomallei* L-asparaginase is effective in killing human leukemia cells, THP-1, mostly due to the deamination of the nonessential amino acid L-asparagine to L-aspartic, thus diminishing the asparagine pool, according to our findings. Even though L-asparagine is a non-essential amino acid, some leukemia and cancer cells get addicted to it for two reasons. First, L-asparagine is essential for the synthesis of glycoproteins and other cellular proteins; second, these cells have low levels of L-asparagine, the counteracting enzyme, resulting in malnutrition and eventually the death of malignant cells. The increase of asparagine and glutamine synthetase, as well as glutamine transporters, which are associated with resistance in vitro [47], could explain the higher concentration of recombinant *B. pseudomallei* L-asparaginase that exhibited IC_50_ on breast cancer MCF-7 (18 IU) cell lines. Other researchers have found that asparagine mRNA, protein, and activity levels in acute lymphoblastic leukemia patients vary greatly [48] and that they are not always linked to in vitro resistance to the drug L-asparaginase. As a result, in addition to asparagine regulation, there may be another mechanism of resistance to L-asparaginase.

Microbial L-asparaginase is an important component of juvenile acute lymphoblastic leukaemia, and finding the L-ASNase with the optimal clinical features is a difficult task. Toxicities associated with treatment necessitate appropriate management, the constant need for novel enzyme sources, and the advancement of existing products.

Overexpression, purification, and characterization of recombinant *B. pseudomallei* L-asparaginase with considerable selectivity for L-asparagine without glutaminase activity were demonstrated in this study. On human leukemia THP-1, HepG2, and MCF-7 cell lines, the recombinant enzyme produced cytotoxicity. As a result, the recombinant *B. pseudomallei* L-asparaginase could be a promising alternative enzyme for the therapy of acute lymphoblastic leukemia, but more research is needed to determine its immunogenicity and toxicity. However, the potential for new anti-leukemic drugs that this investigation may uncover is likely to be substantial.

## Figures and Tables

**Table 1:** *Burkholderia pseudomallei* L-asparaginase deduced amino acid homology with other organisms.

Organism	% Identity	Accession No.
*Burkholderia pseudomallei 1710b*	99.71	ABA50799.1
*Burkholderia pseudomallei*	99.42	WP_122827724.1
*Burkholderia* sp. *BDU5*	92.80	WP_059471291.1
*Burkholderia savannae*	94.24	WP_059642986.1
*Burkholderia mallei*	99.39	WP_073699671.1
*Burkholderia thailandensis*	94.24	WP_009890691.1
*Burkholderia oklahomensis*	93.37	WP_010103079.1
*Trinickia dinghuensis*	80.60	WP_115537086.1
*Burkholderia plantarii*	79.53	WP_198251910.1
*Burkholderia ubonensis*	79.41	WP_060229620.1
*Paraburkholderia terricola*	75.79	WP_073426943.1
*Burkholderia plantarii*	79.24	WP_042625236.1
*Burkholderia glumae*	78.65	QJW77861.1
*Burkholderia ubonensis*	79.41	WP_059987554.1
*Pseudomonas aeruginosa PAO1*	44.12	NP_250028.1
*Saccharomyces cerevisiae S288C*	34.32	NP_010607.3
*Clostridioides*	31.31	WP_003431031.1
*Streptococcus pneumoniae*	32.82	WP_001124778.1
*Mycobacterium tuberculosis H3*	40.57	NP_216054.1
*Deinococcus radiodurans*	36.21	WP_034350512.1
*Escherichia coli O157:H7 str.*	31.42	NP_310501.1
*Bacillus subtilis* subsp.	28.85	NP_390239.1
*Shewanella oneidensis*	29.63	WP_011072398.1
*Caenorhabditis elegans*	27.93	NP_506049.1
*Dictyostelium discoideum AX4*	26.43	XP_645400.1
*Neisseria meningitidis*	28.10	WP_002229812.1

**Table 2:** Purification of *Burkholderia pseudomallei* L-asparaginase.

Purification step	Volume (ml)	Total protein (mg)	Total activity (U)	Specific activity (U/mg)	Yield(%)	Purification fold
Crude extract	50	381	786,890	2065.33	100	1.00
Glutathione Sepharose 4B	10	8. 4	126,014	15,001.67	16.01	7.26

**Table 3:** The effect of reducing agents, EDTA, and certain metal ions (chloride and sulfate forms) on the activity of *Burkholderia pseudomallei* L-asparaginase.

Effector	Residual Activity (%)	

Control	100%	

	1 mM	5 mM
EDTA	60.7	41.2
DDT	81.3	80.6
2-C_2_H_5_SH	97.7	95.2
NaCl	112.5	91.7
KCl	108.4	92.8
HgCl	22.1	14.8
CaCl_2_	84.6	73.4
CuCl_2_	81.8	75.7
MgCl_2_	93.2	88.5
ZnCl2	84.4	80.1
Na_2_SO_4_	88.6	74.9
CuSO_4_	66.4	57.8
MgSO_4_	59.7	48.2
NiSO_4_	77.3	62.4
